# A Quest to Identify Prostate Cancer Circulating Biomarkers with a Bench-to-Bedside Potential

**DOI:** 10.1155/2014/321680

**Published:** 2014-03-12

**Authors:** Jaspreet Singh Batra, Swati Girdhani, Lynn Hlatky

**Affiliations:** Center of Cancer Systems Biology, GeneSys Research Institute, Tufts University, School of Medicine, 736 Cambridge Street, SEMC-CBR112, Boston, MA 02135, USA

## Abstract

Prostate cancer (PCA) is a major health concern in current times. Ever since prostate specific antigen (PSA) was introduced in clinical practice almost three decades ago, the diagnosis and management of PCA have been revolutionized. With time, concerns arose as to the inherent shortcomings of this biomarker and alternatives were actively sought. Over the past decade new PCA biomarkers have been identified in tissue, blood, urine, and other body fluids that offer improved specificity and supplement our knowledge of disease progression. This review focuses on superiority of circulating biomarkers over tissue biomarkers due to the advantages of being more readily accessible, minimally invasive (blood) or noninvasive (urine), accessible for sampling on regular intervals, and easily utilized for follow-up after surgery or other treatment modalities. Some of the circulating biomarkers like PCA3, IL-6, and TMPRSS2-ERG are now detectable by commercially available kits while others like microRNAs (miR-21, -221, -141) and exosomes hold potential to become available as multiplexed assays. In this paper, we will review some of these potential candidate circulating biomarkers that either individually or in combination, once validated with large-scale trials, may eventually get utilized clinically for improved diagnosis, risk stratification, and treatment.

## 1. Introduction

In 2014, more than 200,000 American men will be diagnosed with prostate cancer (PCA). It is the most frequently diagnosed solid tumor and the second leading cause of cancer-related deaths amongst men in the United States. It is estimated to cause 28% of the total number of cancer cases and 10% of the total cancer deaths amongst adult male cancer patients. One in 6 men carries a lifetime risk of a PCA diagnosis [1]. Prostate-specific antigen (PSA) is the biomarker currently being used for PCA. PSA-based screening test has been proved to be a useful prognostic tool. Usually high preoperative values have been related to advanced disease and a poorer clinical outcome. Clinicians initially used PSA for monitoring PCA patients after treatment to detect disease progression, treatment failure, or potential relapse [2] and then subsequently recommended its use for screening purposes [3–5]. Since the late 1980s, the introduction of PSA screening along with digital rectal exam (DRE) and transrectal ultrasound (TRUS) in general clinical practice has led to an increase in the documented incidence of PCA [6, 7]. This trend has been accompanied by an increase in invasive procedures with radical prostatectomy rates nearly six-fold higher in 1990 than in 1984 [8]. While aggressive screening practices in the US resulted in patients getting diagnosed at a much earlier and potentially more curable stage of the disease [2–4], it was followed by widespread criticism of overdiagnosis and overtreatment of indolent tumors. A recent analysis done by Draisma et al. (2009) using different simulation models estimated the overdiagnosis rate for screen detected cancers that otherwise would have never been diagnosed in the absence of screening test to be 23% and 42% [9]. Similarly, Welch et al. estimated the magnitude of overdiagnosis from randomized trials to be 60% for PSA detected PCA [10].

Even though PSA is currently being used as a marker for diagnosis, its values are now being recognized as representing the relative degree of risk for PCA. At 4 ng/mL (upper limit of the reference interval) PSA fails to detect a substantial number of cancers, and the Prostate Cancer Prevention Trial has concluded that there is no cutoff value for PSA level with simultaneous high sensitivity and high specificity for monitoring healthy men for PCA but rather a continuum of prostate cancer risk at all values of PSA [11]. The controversy surrounding the use of this marker is currently being debated, because it is still unclear whether PSA screening has truly led to a decline in mortality due to PCA [12, 13]. Under normal conditions, the intact architecture of prostate gland keeps PSA tightly confined and only low levels of PSA can be detected in blood. The increase in serum PSA in prostate cancer cannot be explained by increased PSA expression; instead it may represent abnormalities in prostate gland architecture and vascularization, although the exact mechanism is unclear [14]. PSA screening test carries a sensitivity and specificity in the range of 70% to 90% and 20% to 40%, respectively [15]. The area under the curve (AUC) of the receiver operating characteristic (ROC) curve is between 0.55 and 0.70 for the ability of PSA to identify patients with PCA [15]. It is not specific for PCA and more commonly is elevated in noncancerous events such as infections, trauma, benign prostatic hyperplasia (BPH), and growth in prostate volume. The positive predictive value for PSA-based screening for PCA is between 25% and 40% [16], with patients in the gray zone of 4–10 ng/mL having a 25% chance of harboring latent PCA and about 15% of men with PSA concentrations of <4 ng/mL also displaying PCA [17]. These shortcomings of PSA as a marker have created a necessity to search for novel markers of PCA to better predict disease occurrence, progression, and final outcome as well as avoid overtreatment of latent tumors.

This review focuses on those upcoming circulating biomarkers that are being evaluated for their diagnostic, prognostic, therapeutic, and predictive properties. Other biomarkers that in recent times have generated considerable interest such as microRNAs, DNA methylation, exosomes, and platelet sequestered biomarkers specific to PCA are also briefly discussed.

The National Cancer Institute defines a biomarker as a biological molecule found in blood, other body fluids, or tissues that is a sign of a normal or abnormal process or of a condition or disease. A biomarker may be objectively measured and independently validated. The level or expression of a biomarker should not only be sensitive and specific to a particular disease (or natural process) but also correlate with the progress of the disease or its response to a treatment [18]. To fulfill all of these criteria there is a general consensus that a panel of biomarkers is required rather than an individual biomarker for a particular condition/disease [19–23].

Cancer biomarkers can play a crucial role in disease diagnosis and in predicting its outcome. They can potentially provide vital information to determine whom to treat aggressively and whom to follow up with active surveillance. They may also predict who will respond and how much response to expect with the established treatment modalities for a particular cancer [24]. Lastly, they may shed some light onto the development of newer, safer, and more efficacious therapies [24–26].

For the purpose of this review we will concentrate on the circulating biomarkers present in bodily fluids that can be evaluated by using either minimally invasive procedures (blood) or noninvasive procedures (urine) rather than tissue markers. Development of a panel of sensitive and specific circulating biomarkers would lead to a tremendous reduction in unnecessary biopsies and will have an added advantage of being repeated at regular intervals and can be utilized for follow-up assessment after radical prostatectomy, radiotherapy, hormone therapy, or chemotherapy.

**Figure 1:**
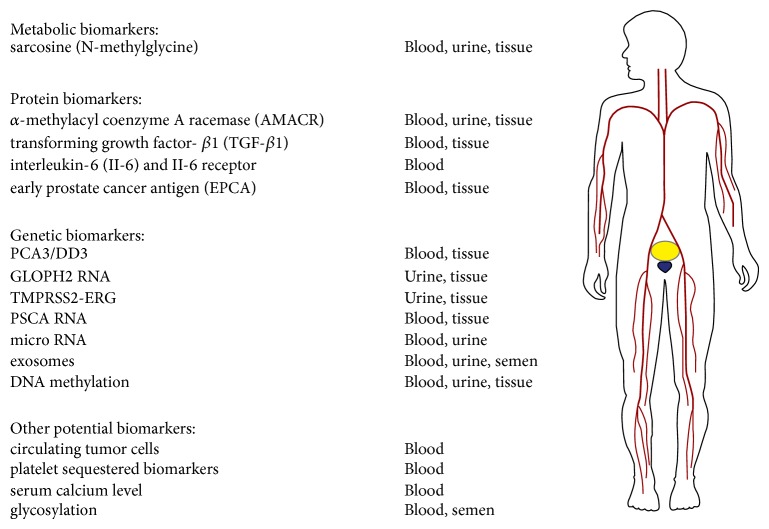
Summary of prostate cancer biomarkers (as discussed in this paper) that hold the potential to be implemented in clinical practice in the near future. The corresponding sample sources that may be utilized for regular testing of these biomarkers are also listed.

For the purpose of this review paper, candidate circulating biomarkers have been subdivided as (1) circulating metabolic biomarkers, (2) circulating protein biomarkers, (3) circulating genetic/epigenetic biomarkers, and (4) other potential biomarkers (Figure 1).

## 2. Circulating Metabolic Biomarker

### 2.1. Sarcosine

Sarcosine (N-methylglycine) is a natural amino acid that is found in muscle and other body tissues. It was shown to induce invasive phenotype in benign prostate epithelial cell and, when detected in urine, it may be used as an indicator of malignant prostate cancer [27]. A difference between benign prostate, clinically localized prostate cancer, and metastatic prostate cancer was also demonstrated based on the levels of sarcosine in urine, blood, and tissues of each subgroup [27]. Attenuation in cell invasion was observed in DU145 prostate cancer cells after knocking down glycine-N-methyl transferase, the enzyme that catalyzes the production of sarcosine from glycine attenuated prostate cancer invasion. While addition of exogenous sarcosine or knocking down of sarcosine dehydrogenase, the enzyme responsible for sarcosine degradation stimulated an invasive phenotype in primary benign prostate epithelial cells. Some experts in this field have pointed to the limitations to these findings, namely, small sample size and the need for independently confirming these results in larger cohorts of patients [28]. Ever since these preliminary results were published there have been studies that either challenged [29] or supported [30] the above findings, While others have concluded that neither of these contrasting studies have used validated analytical method to measure sarcosine in urine [31, 32]. Because of these equivocal findings it is yet to be determined whether sarcosine may play a vital role to promote prostate cancer cells toward invasion and aggressiveness [33]. Given the inconclusive results, it is clear that further investigations are needed to determine the role of sarcosine in prostate cancer progression and invasion as well as to establish its potential as a prostate cancer biomarker.

## 3. Circulating Protein Biomarkers

### 3.1. *α*-Methylacyl Coenzyme A Racemase (AMACR)

*α*-Methylacyl coenzyme A racemase (AMACR) is an isomerase family enzyme primarily located in mitochondria or peroxisomes. AMACR overexpression as identified by immunostaining has been reported to be a diagnostic indicator of PCA and other solid tumors [34, 35]. It has become established as a tissue biomarker for PCA [36] and currently AMACR detection in PCA biopsy samples is regarded as an improvement over the serum PSA test. Its development as a circulating marker has gained prominence lately. AMACR mRNA has also been detected in prostatic secretions obtained from postmassage urine specimens. When AMACR transcripts (mRNA) were normalized to PSA transcripts it was shown to be predictive of PCA [37]. On the contrary, another study [38] contradicted these findings by conducting a multiplex study of urine markers. Autoantibodies against this protein have also been found in serum and a study from 2004 [39] suggests that it could distinguish cancerous from benign blood samples better than PSA.

### 3.2. Transforming Growth Factor-*β*1 (TGF-*β*1) and Interleukin-6 (IL-6)

Transforming growth factor-*β*1 is a growth factor that is involved in a wide variety of cellular mechanisms including, but not limited to, cell proliferation, differentiation, immune response, and angiogenesis [40]. Its increased local expression in PCA has been related to higher tumor grade, local invasion, distant metastasis, and biochemical recurrence [41]. In a study by Ivanovic et al. [42], an immunoassay was used to measure preoperative plasma levels of TGF-*β*1 in PCA patients. The study correlated increased levels of TGF-*β*1 with invasive PCA. While Shariat et al. has shown TGF-*β*1 association with extracapsular extension, seminal vesicle invasion, and biochemical recurrence [43, 44]. Further large scale level studies are needed to validate these findings that can serve to determine its use as a biomarker for disease progression.

Interleukin-6 (IL-6) is a cytokine secreted by a variety of cell types with variable effects on immune and hematopoietic mechanisms [45]. Both in vitro and in vivo studies have shown increased expression of IL-6 and its soluble receptor (IL-6R) in PCA cells [46] and tissue [47]. Michalaki et al. and Nakashima et al. reported that elevated levels of IL-6 and its receptor in serum are associated with metastatic and hormone refractory disease [48, 49]. Based on these findings, Kattan et al. [50] validated and enhanced the prognostic ability of an existing preoperative nomogram by adding plasma levels of TGF-*β*1 and soluble IL-6R from samples collected prior to radical prostatectomy. A multi-institutional dataset of 423 PCA patients treated with radical prostatectomy validated these results [21], suggesting a potential role for TGF-*β*1 and soluble IL-6R to improve risk stratification of biochemical recurrence after radical prostatectomy and help in guiding clinicians to identify patients who need aggressive follow-up.

### 3.3. Early Prostate Cancer Antigen (EPCA)

Early prostate cancer antigen is a nuclear matrix protein, originally discovered in rat prostate tissue [51]. EPCA is linked to nuclear transformations that occur in early stages of prostate cancer development [52]. Based on immunohistochemical staining, it has been reported by different groups [52, 53] to be present in various cancer precursor lesions in addition to prostate cancer tissue. More recently studies [54, 55] using EPCA-based enzyme linked immunosorbent assay have provided substantial data to confirm the potential diagnostic value of serum EPCA.

## 4. Circulating Genetic Biomarkers

### 4.1. PCA3/DD3

Prostate cancer antigen 3 (PCA3) also known as differential display code 3(DD3) is a noncoding mRNA, which is specifically produced by prostate gland. PCA3 has been found to be highly overexpressed in malignant prostate tissue in comparison with benign prostate tissue [56, 57] and its yield is improved in urine specimen if preceded by attentive DRE/prostate massage. A robust urine test (Hologic Gen-Probe's PROGENSA PCA3 Assay) became commercially available in 2006. Its role in predicting tumor volume [58, 59] or extracapsular extension on final pathology after prostatectomy [59] has been established. Since it has been reported that PCA3 holds less sensitivity but high specificity for PCA than PSA [60–62], perhaps combining PCA3 with PSA or other new biomarkers like AMCAR will improve the sensitivity [63] and help better stratify the patients for specific treatment decisions.

### 4.2. GLOPH2 RNA

Golgi phosphoprotein 2 (GOLPH2), also named as GP73, is a 73 kDa Golgi apparatus associated protein that is coded by the GOLM1 gene located on Chromosome 9q21.33. Various epithelial cells are reported to express this protein. Besides PCA, it is also overexpressed in hepatocellular carcinoma and adenocarcinoma of the lung [64–66]. While it is still unclear what the exact functions and mechanics of GLOPH2 regulation are, work by Kristiansen et al. [64] suggest that it may be involved in posttranslation protein modification, cell signaling regulation, transport of secretory proteins, or maintenance of Golgi apparatus function [64]. A recent study by Laxman et al. [38] identified increased urinary GOLPH2 transcriptome along with SPINK1, PCA3, and TMPRSS2-ERG as a significant predictor of PCA. Given the lack of additional data, it is clear that further studies are required to determine the appropriate diagnostic or prognostic value of circulating/excreted GOLPH2 in PCA.

### 4.3. Urinary TMPRSS2-ERG

About 90% of gene fusions in prostate cancer are accounted by the fusion between the transmembrane protease, serine 2 (TMPRSS2), that is a strong androgen-regulated gene and the ERG gene, a v-ets erythroblastosis virus E26 oncogene homolog (avian). The ERG gene belongs to the ETS family of transcription regulators, which contributes to carcinogenesis and tumor progression [67]. These gene fusions are presumed to result in overexpression of ETS transcription factors under the control of androgen response elements [68]. Some studies [69, 70] have already investigated the presence of TMPRSS2-ERG mRNA in PCA patients' urine samples. Since it is absent in about 50% of PCA cases [69], its use lies in combined assays with other biomarkers, such as PCA3. Tomlins et al. [69] evaluated 1312 prospectively enrolled subjects and established that urine TMPRSS2-ERG along with PCA3 enhanced serum PSA predicted PCA risk and clinically relevant cancer on biopsy. In addition, urine TMPRSS2-ERG levels seem to be associated with indicators of clinically significant prostate cancer at biopsy and prostatectomy such as Gleason score, the percent of tumor observed, and number of cores with tumor. In the absence of any ongoing or recently published trials, these biomarkers (TMPRSS2-ERG & PCA3) can at the best be used as an adjunct to PSA. In addition, urinary mRNA for TMPRSS2-ERG or PCA3 is measured relative to PSA mRNA in urine, thus dictating the dependency of these tests on any fluctuation in the levels of urinary mRNA for PSA.

### 4.4. Prostate Stem Cell Antigen (PSCA)

Prostate stem cell antigen (PSCA) is a glycosylphosphatidylinositol-anchored cell surface antigen. It has been identified in the epithelium of several organs, such as the prostate, stomach, bladder, and gallbladder [71–73]. PSCA was detected in PCA tissues by immunohistochemistry, and PSCA RNA was found in blood samples of patients diagnosed with PCA. Increased PSCA production was correlated with an increased risk of PCA, a higher Gleason score, a higher stage, seminal vesicle invasion, capsular invasion, and the presence of metastasis [71, 74–76]. It was observed to be jointly amplified with c-myc in locally advanced prostate cancers [77, 78]. When compared with the mRNA of other circulating prostate markers like PSA and PSMA, PSCA displayed inferior sensitivity and considerable inability to distinguish between malignant and benign disease, though its disease specificity and independent predictive value were the highest [79]. By using human PSCA transfected PC3 cell lines and inoculating subcutaneously in female NCR nude mice, researchers had shown that anti-PSCA monoclonal antibodies inhibited tumor growth and metastasis formation [80]. It was postulated to be a potential therapeutic target for immunotherapeutic procedures [81–83]. Despite these significant findings there are still no definitive conclusions regarding its use in clinic as a serum biomarker for PCA. Need for additional data as well as validation and reproducibility of the techniques to quantify the serum levels [84] are some of the shortcomings that need to be addressed before PSCA can be considered as a valuable biomarker for further development. More studies are awaited to further evaluate and determine its effectiveness as a clinical prostate cancer marker.

### 4.5. Micro RNA

MicroRNAs (miRNAs) are small endogenous single stranded, noncoding RNA molecules of approximately 17- to 27-nucleotide length. Though the majority of miRNA resides intracellularly, stable miRNA has been observed in extracellular body fluids including blood and urine. miRNAs seem to play an important role in modulating immune response, DNA repair, apoptosis, oxidative stress, carcinogenesis, and cancer progression [85]. Extracellularly, miRNAs have been implicated to play an important role in distant signaling by modifying gene expression. They tend to negatively regulate the targeted mRNAs at the posttranslational level by binding with imperfect complementarities to the sites within the 3′ untranslated region (UTR) of these mRNAs. In this manner, they are able to reduce the stability and translational efficiency of target mRNAs. Like protein-coding RNAs, miRNAs have the potential to either promote (oncomirs) or inhibit (tumor suppressor miRNAs) cancer [86, 87]. A single miRNA may target more than 200 different mRNAs and, vice versa, a particular target could be regulated by different miRNAs [88]. So far, close to 1400 human miRNAs have been identified.In a recent study by Agaoglu et al. [89] miR-21 (AR-regulated miRNA) and miR-221 were elevated in the plasma of men with localized PCA compared with healthy controls. In addition, miR-141 along with miR-21 and miR-221 were increased in samples from men with bone metastases compared with men with localized/locally advanced disease, and miR-141 could accurately distinguish between these groups (AUC = 0.755). The oncogenic properties of miR-221 have been attributed to its control of cyclin dependent kinase (Cdk) inhibitors p27KIP1 and p57KIP2, effectively controlling the G1-to-S phase transition [90, 91] as well as PI3K and PTEN signaling [92]. Likewise, miR-21's oncogenic effects and drug resistance properties have been attributed to its control of downstream target, programmed cell death 4 (PDCD4) [93, 94]. Shen et al. also confirmed that miR-20a, miR-21, miR-221 were differentially expressed based on PCA stage or risk assessment by Cancer of the Prostate Risk Assessment (CAPRA) or D'Amico scores [95]. Other studies using tissue-based microarrays and PCR tests have reported conflicting results showing loss of miR-221 and miR-222 either during the early stage of cancer [96] or during the aggressive stage [97, 98]. Such conflicting reports indicate the need for further larger scale investigations and also hint at the idea that expression levels of miRNAs might be differentially controlled at different stages of the PCA progression.

Bryant et al. [99] has identified 12 differentially quantitated plasma miRNAs between cohorts of PCA patients and healthy men with miR-107 showing the highest fold-change. Five of the miRNAs were also detected in urine and miR-107 and miR-574-3p were present at significantly higher concentrations in urine samples from men with PCA compared with healthy controls. The authors also went on to identify 16 miRNAs, including miR-141, miR-200b, and miR-375, at differential levels in the plasma of PCA men with either localized or metastatic disease. Another group (Nguyen et al.) reconfirmed similar findings by demonstrating that elevated expression of circulating miR-375 and miR-141 can distinguish patients with metastatic castration-resistant prostate cancer (CRPC) from those with low-risk, localized prostate cancer [100].

miR-143, miR-145, and miR-200 family miRNAs have been identified as tumor suppressor miRs and are known to be involved in epithelial-mesenchymal transition (EMT) either by loss of their suppression on EGFR/RAS/MAPK pathway or by targeting ZEB1 and ZEB2 [101]. In a study by Peng et al. [102], both miR-143 and miR-145 expressions were significantly decreased in PCA and were negatively associated with metastasis. Based on all these observations, circulating miRNAs profiling offers the potential to improve the diagnosis of cancer and might predict outcome for cancer patients. Nevertheless, further studies are required to better understand the function of these potential biomarkers and their relation with the development, progression, and spread of PCA.

### 4.6. Exosome

Exosomes are membrane-bound nanoparticles (30–150 nm) normally released from cells in the body that contain molecules such as proteins, mRNA, and miRNA [103]. These microvesicles are generated from internalized parts of the cellular membrane and subsequently secreted into bodily fluids such as blood, urine, or semen. They may contain varying proportions of functional RNA, microRNA, and proteins. Increased level of exosomes was detected in the serum from prostate cancer patients compared to men with no disease, and elevated levels of exosomes may also correlate with disease aggressiveness [104]. They may act as messengers and may play a crucial role in cell-cell interaction both in vicinity and at distance. Exosomes are constituents of urine, with some variability amongst different patients' urine samples. Such vesicles may be a useful noninvasive source of markers for prostate cancer since they often carry genetic components that come directly from tumors. A recent review highlights the upcoming role of exosomes as potential biomarkers for PCA [105]. Likewise, some recent studies have also reported the presence of PCA3 and TMPRSS2-ERG fusion, two known prostate cancer biomarkers, in exosomes from urine samples of prostate cancer patients [106, 107].

### 4.7. DNA Methylation

Cancer phenotypes have a complex and heterogeneous character, which cannot be explained by genetic defects alone [108]. Several groups have shown the crucial role of epigenetic modifications in the manifestation of various cancer types [109–112]. Epigenetic modifications are defined as heritable changes in the expression and regulation of gene expression without altering the DNA sequencing [113].

Feinberg and Vogelstein first reported aberrant DNA methylation as an epigenetic event to be associated with cancer as a consequence of the alteration it causes in normal gene regulation [114]. Many investigators have identified the role of DNA methylation in the development and progression of PCA [115, 116]. Aberrant methylation may include hypermethylation, hypomethylation, or loss of imprinting. Hypermethylation refers to gain of methylation at specific sites, which under normal conditions are unmethylated. This happens mainly at promoter CpG islands (CGIs), which are defined as a DNA sequence (>2K base pairs) with a GC content greater than 50% and an observed : expected ratio of more than 0.6 [117, 118]. The promoter CGI hypermethylation in turn is associated with stabilization of transcriptional repression and loss of gene function and mostly occurs in tumor suppressor genes [119, 120]. Hypermethylation of glutathione S-transferase-pi gene (GSTP1) promoter has been reported to be the most frequent epigenetic modification in PCA and is present in 70% of high-grade prostatic intraepithelial neoplasia (high-grade PIN) lesions and 90% of cancerous tissue samples when compared to normal or benign hyperplastic epithelium [121]. GSTP1 methylation patterns can also be detected in serum and urine making the procedure less invasive and the circulating biomarker a potential candidate for clinical use. It has a high specificity (86.8–100%) but low sensitivity both in urine (18.8–38.9%) and serum/plasma (13.0–72.5%) [122–124]. To improve upon the utilization of this biomarker Rouprêt et al. (2007) showed that the promoter methylation pattern of four genes, GSTP1, RASSF1a, RARbeta2, and APC, was able to differentiate malignant from nonmalignant cases with 86% sensitivity and 89% specificity [125]. Similarly in 2005, Hoque et al. had examined promoter methylation patterns of nine genes in urine sediment to differentiate PCA patients from normal. Results from urine samples correlated with methylation patterns reported from corresponding primary tumor tissues. Out of the 9 genes, just the four-gene combination (p16, ARF, MGMT, and GSTP1) was able to detect prostate cancer with 87% sensitivity and 100% specificity [122]. Another study by Ellinger et al. (2009) utilized serum samples for methylation status using another 4-gene combination (GSTP1, PTGS2, RPRM, and TIG) that provided slightly higher specificity (AUC = 0.699) when serum samples from PCA patients were compared with BPH patients [126].

A recent review article on methylation markers for PCA concludes that evidence on the prognostic utility is still inconclusive and recommends further research with larger sample sizes, and adequate follow-up data and to include patients from other races/ethnicities as well as those who have received treatment other than radical prostatectomy [127].

## 5. Other Potential Biomarkers

### 5.1. Circulating Tumor Cells

Circulating tumor cells (CTCs) were first discovered in 1869 [128]. In recent times they have been at the center of intense scientific investigation since they circulate in blood and are postulated to mediate hematogenous metastasis. They may potentially provide beneficial information for risk stratification, to gauge therapy response for better clinical management of cancer patients, predict disease recurrence, and provide new insights for an individual cancer treatment strategy [129]. In addition, CTCs have been shown to manifest bidirectional flow and are reported to colonize their tumors of origin. This behavior is known as “tumor self-seeding” and is considered to accelerate primary tumor growth, angiogenesis, and stromal recruitment [130]. These cells may also be a source of molecular information, such as TMPRSS2-ERG, AR, and PTEN copy number status [131]. Some studies have reported that the increased number of CTCs in the blood of castration-resistant prostate cancer (CRPC) patients predicts a less favorable survival outcome [132, 133]. Despite these promising findings, detecting CTCs and extracting their molecular information are both labor-intensive and expensive. In addition, these cells may evade the detection through a phenotypic switching mechanism such as EMT-like process. Ongoing clinical trials may be proven to be helpful in establishing CTCs as potential surrogate markers for this disease in the near future.

### 5.2. Platelet Sequestered Biomarkers

A recent study by Suades et al. [134] has revealed that platelet-derived microparticles (pMPs) enhance platelet deposition and thrombus formation in atherosclerotic arteries as well as in normal blood conditions. They also report that these microparticles have functional effects on cardiovascular atherothrombotic disease. In 2010, Kerr et al. [135] described that murine platelets sequestered functionally active molecules like monocyte chemotactic protein-1 (MCP-1) and tumor necrosis factor-*α* (TNF-*α*) in blood collected from a xenograft model injected with human prostate cancer cells. The author also reported preferential localization of tumor-derived MCP-1, matrix metalloproteinase-2 (MMP-2), receptor activator of NF-kappaB (RANK), receptor activator of NF-kappaB ligand (RANKL), and tissue inhibitor of matrix metalloproteinase-1 (TIMP-1) and host-derived TNF-*α* and thrombopoietin (TPO) within host platelets. Similarly, Nilsson et al. [136] demonstrated that (mutant) RNA from tumor cells could be transferred into blood platelets both in vitro and in vivo. They also showed that the cancer associated RNA biomarker PCA3 was preferentially sequestered in the circulating blood platelets of PCA patients.

These findings suggest that protein and/or genetic elements sequestered within host platelets act as messengers for tumor cells and may be potential biomarkers for PCA diagnosis and its progression.

### 5.3. Serum Calcium Level

Nearly fifteen years ago Lin et al. [137] reported increased in vitro proliferation due to decreased apoptosis and increased cell attachment of skeletal metastatic prostate cell lines (PC-3 and C4-2B) in the presence of elevated serum calcium (Ca^2+^). The proliferative effect of elevated serum calcium was associated with higher expression of the calcium-sensing receptor, which is a membrane-bound, heterotrimeric G-protein-coupled receptor that transduces signals involved in serum calcium regulation. Recently, Schwartz [138] reported that prostate cancer patients with increased serum calcium levels (total and/or ionized) or any factor that leads to it (such as high serum parathyroid hormone) carry an increased risk of mortality. These results were derived from the National Health and Nutrition Examination Surveys III (NHANES) and reaffirmed findings from their 2007 study where a greater than 2.5-fold increased risk amongst men was found in the highest tertile of serum calcium [139]. Another group tried to determine a serum calcium level association with biochemical recurrence (BCR) following salvage radiation therapy; however, no evidence of a linear association between serum calcium and BCR was identified [140].

These findings suggest that serum calcium (possibly in combination with other biomarkers) may be useful as a prognostic tool to identify patients with higher risk of mortality from PCA rather than serving as diagnostic tool. Further molecular and epidemiological investigations are required to better understand the significance of serum calcium levels as a promising prospective biomarker for PCA.

### 5.4. Glycosylation

Glycosylation is one of the most common protein posttranslational modifications. It is a template-free process carried out by various enzymes like transferases and endoglycosidases. It involves attachment of various glycan moieties (fucose, mannose, sialic acid, etc.) to different proteins and lipids before undergoing further intricate pruning and addition of several monosaccharides. Aberrant glycosylation has been associated with many diseases including multiple types of cancers [141–143]. Some studies have suggested correlations between glycan structures and cancer prognosis [144, 145]. There are three distinct types of protein glycosylations: N-glycans, O-glycans, and glycosaminoglycans. The same protein may possess different glycan structures, referred to as glycoforms.

In 2007, Kyselova et al. [146] utilized matrix-assisted laser desorption/ionization mass spectrometry (MALDI-MS) profiles derived from sera of PCA patients (*n* = 24) and disease-free group (*n* = 10). They demonstrated that when these samples from PCA patients were compared to disease-free group out of the fifty N-glycan structures that were observed ten were significantly more and two were significantly less present and the differences were statistically significant (ANOVA scores < 0.001). Similarly, in 2008 de Leoz et al. profiled N-glycan in human sera from twenty PCA patients (ten under active surveillance and ten after radical prostatectomy) and in immortalized pRNS prostate epithelial cell lines (pRNS) that express wild type or mutant androgen receptors [143]. While data from ex vivo experiments was not conclusive, the one from human sera reported that fourteen glycans were downregulated and ten were upregulated in the active surveillance group when compared to post-radical-prostatectomy group.

Various groups have also studied glycosylated PSA as a biomarker for PCA. When PCA patients' sera were compared to BPH patient's sera, an increased core fucosylation and an increased expression of *α*2-3 linked sialic acid in PCA serum glycomes were observed [147]. Similarly, decreased sialylation was observed in seminal fluid samples from PCA patients when compared to control group [148] and BPH group [149].

Major advancements in mass spectrometry and separation have tremendously helped characterize glycan changes brought about by different disease processes. This has helped us better understand these changes and their role in carcinogenesis and progression. Still, more studies are needed to validate these findings and to help us appreciate their precise role in diagnostics.

## 6. Future Directions

Since 1994 PSA testing in prostate cancer has been the primary biomarker used in aggressive screening, early diagnosis, and treatment. However concerns that its high false positive results may create confusion for patients and clinicians like in deciding who and when to treat have motivated a search for more sensitive and highly specific biomarkers. Our current PCA diagnosis and management protocols need significant update, to better address some of the pitfalls left unaddressed by singularly deploying PSA testing. When used in the right context, some of these potential prostate cancer biomarkers could avoid unnecessary biopsies, reduce the number of radical prostatectomies and the requirement for other treatment modalities like radiotherapy, stratify organ-confined tumors (curable by surgery), monitor progression during “watchful waiting,” and/or lower overall mortality from the disease. A more rational approach to biomarker discovery, combined with modern molecular science and bioinformatics, will eventually allow clinicians to better diagnose and target treatment for those patients who are most likely to benefit.
